# Sex-Related Differences in Metastatic Melanoma Patients Treated with Immune Checkpoint Inhibition

**DOI:** 10.3390/cancers14205145

**Published:** 2022-10-20

**Authors:** Ken Kudura, Lucas Basler, Lukas Nussbaumer, Robert Foerster

**Affiliations:** 1 Department of Nuclear Medicine, University Hospital Zurich, 8091 Zurich, Switzerland; 2 Institute of Radiooncology, Cantonal Hospital Aarau, 5001 Aarau, Switzerland; 3 Faculty of Medicine, University of Zurich, 8091 Zurich, Switzerland; 4 Institute of Radiooncology, Cantonal Hospital Winterthur, 8400 Winterthur, Switzerland

**Keywords:** Positron Emission Tomography Computed Tomography, melanoma, immunotherapy, CTLA-4, PD-1, sex differences, gender medicine

## Abstract

**Simple Summary:**

Immune checkpoint inhibitors, ICI, have revolutionized the treatment of advanced melanoma. However, given the small number of patients responding to immunotherapy and the high risk for immune-related adverse events, there has been a rising interest in recent publications to identify factors that influence response to immunotherapies, including sex. We aimed at investigating sex-related differences in patients with advanced melanoma treated with ICI by linking the assessment of inflammatory response in peripheral blood, onset of IRAEs during therapy and treatment response in short- and long-term. Men with advanced melanoma showed a significantly better response to immunotherapy in short- and long-term than women. Higher immune activation in peripheral blood before and after initiation ICI might be linked to favorable treatment response during and after ICI in favor of men and decoupled from the onset of IRAEs. Given the significantly higher immunotoxicity and worse outcome experienced by women compared to men the use of ICI should be chosen carefully in women with advanced melanoma.

**Abstract:**

Objectives: We aimed to investigate sex-related differences in patients with advanced melanoma treated with ICI by linking the assessment of inflammatory response in peripheral blood, onset of immune-related adverse events IRAEs during therapy and treatment response in short- and long-term. Methods: For the purpose of this single-center retrospective study metastatic melanoma patients treated with ICI were included. Baseline patient characteristics, blood sample tests and the onset of immune-related adverse events IRAEs were documented based on clinical records. The short-term treatment response was assessed with ^18^*F*-2-Fluor-2-desoxy-D-glucose Positron Emission Tomography/Computed Tomography FDG-PET/CT scans performed six months after initiation of ICI. The overall survival OS and progression-free survival PFS were used as endpoints to assess the long-term response to immunotherapy. Results: In total, 103 patients with advanced melanoma (mean age 68 ± 13.83 years) were included, 29 women (mean age 60.41 ± 14.57 years) and 74 men (mean age 65.66 ± 13.34 years). The primary tumor was located on a lower extremity in one out of three women and on the head/neck in one out of three men (*p* < 0.001). While the superficial spreading (41%) and nodular (36%) melanoma subtypes represented together 77% of the cases in male population, women showed a more heterogenous distribution of melanoma subtypes with the superficial spreading (35%), nodular (23%), acral lentiginous (19%) and mucosal (12%) melanoma subtypes being most frequent in female population (*p* < 0.001). Most differences between women and men with regards to inflammatory parameters were observed six months after initiation of ICI with a higher median NLR (*p* = 0.038), lower counts of lymphocytes (*p* = 0.004) and thrombocytes (*p* = 0.089) in addition to lower counts of erythrocytes (*p* < 0.001) and monocytes (*p* < 0.001) in women towards men. IRAEs were more frequent in women towards men (*p* = 0.013). Women were more likely to display endocrinological IRAEs, such as thyroiditis being the most frequent adverse event in women. Interestingly IRAEs of the gastrointestinal tract were the most frequent ones in men. Finally, men with advanced melanoma showed a significantly better response to immunotherapy in short- (*p* = 0.015) and long-term (OS *p* = 0.015 and PFS *p* < 0.001) than women. In fact, every fourth man died during the course of the disease, while every second woman did not survive. (*p* = 0.001). Conclusion: Men with advanced melanoma showed a significantly better response to immunotherapy in short- and long-term than women. Higher immune activation in peripheral blood before and after initiation ICI might be linked to favorable treatment response during and after ICI in favor of men and decoupled from the onset of IRAEs. Given the significantly higher immunotoxicity and worse outcome experienced by women compared to men the use of ICI should be chosen carefully in women with advanced melanoma.

## 1. Introduction

Melanoma is the leading cause of death among skin cancers [1,2]. The incidence of melanoma has significantly increased over the past decades, becoming the most rapidly increasing cancer in predominantly fair-skinned populations [3,4,5].

Immune checkpoint inhibitors (ICI), e.g., monoclonal antibodies targeting cytotoxic T-lymphocyte-associated antigen-4 (CTLA-4), programmed cell death protein-1 (PD-1) and PD-ligand 1 (PD-L1), have revolutionized the treatment of advanced melanoma. However, given the small number of patients responding to immunotherapy (about 40 to 50% of all treated patients) and the high risk for immune-related adverse events IRAEs (observed in up to 60% of all patients), there has been a rising interest in recent publications to identify factors that influence response to ICI including sex [6,7,8,9].

Sex is determined by an organization of reproductive organs leading to different sex steroid levels in women and men responsible for distinct innate and adaptive immune responses between women and men. Different immune responses in men and women result in different risks for autoimmune disease and malignancies. While men are at almost two-fold greater risk of death from malignant disease than women, 80% of autoimmune diseases occur in women [8].

Interesting investigations and reviews onwards the efficacity of ICI in men and women with advanced melanoma have been published in recent years with conflicting results on the prognostic value of sex [10,11,12,13,14,15,16,17].

The prognostic value of IRAEs experienced by melanoma patients treated with ICI has also been subject of recent prospective and retrospective investigations with interesting results suggesting a favorable outcome with the onset of IRAEs [18,19].

Furthermore, there has been a rising interest in prognostic value of inflammatory blood parameters at baseline and at early stage of treatment in recent literature. In this context, neutrophils-to-lymphocytes ratio has been described as promising biomarker that suggests increased lymphocyte count and decreased neutrophil count at early stage of the disease are associated with favorable outcomes [20,21,22,23,24,25,26,27].

In knowledge of the recent literature, we aimed to investigate whether higher immune activation in peripheral blood promotes the onset of IRAEs and a favorable short- and long-term outcome after ICI in men and women with advanced melanoma.

## 2. Materials and Methods

### 2.1. Patient Cohort

The following inclusion criteria were used for the present single-center retrospective study:

(a). The patient had a histopathologically confirmed metastatic melanoma;
(b). The patient was treated either with a single checkpoint-inhibition (anti-PD-1) or dual checkpoint-inhibition (anti-PD-1/anti-CTLA-4);
(c). The patient was treated in the Department of Dermatology of the University Hospital Zurich in Switzerland between 2013 and 2019;

Only patients fulfilling these three criteria were included.

All included patients consented the use of their clinical data for research purposes.

This study was conducted in compliance with Good Clinical Practice GCP-rules and the Declaration of Helsinki (ethics board approval KEK-ZH-Nr: 2014-0193).

For the purpose of our investigations, we focused on four key aspects between male and female patients with advanced melanoma; we wanted to highlight in context of immunotherapy. All clinical data were obtained based on internal clinical records.

### 2.2. Baseline Characteristics

Patient baseline characteristics including sex, age, anatomical site of melanoma metastases, anatomical site of primary tumor, thickness of primary tumor, histopathology of primary tumor, single or dual immune checkpoint inhibition and American Joint Committee on Cancer AJCC stage (7th and 8th edition) were collected. 

#### 2.2.1. Inflammatory Response to ICI in Peripheral Blood

In order to assess patient inflammatory response to ICI in peripheral blood, the following proteins and cells were recorded at baseline, and three and six months after initiation of immunotherapy per patient: basophiles (g/L), c-reactive protein (mg/L), erythrocyctes (per pL), leucocytes (g/L), lymphocytes (g/L), monocytes (g/L), neutrophils (g/L) and thrombocytes (g/L). The neutrophils-to-lymphocytes-ratio NLR was then calculated by dividing the absolute neutrophils count by the absolute lymphocytes count.

The average interval between blood samples at baseline and three months after initiation immunotherapy was 107.1 days and 93.5 days between the time points three and six months after treatment start.

#### 2.2.2. Toxicity of ICI

In order to assess the experienced toxicity of ICI we reported the onset of IRAEs, the number or IRAEs per patient and the type of IRAEs during treatment. 

#### 2.2.3. Patient Response to ICI

- Short-term response to ICI

For this purpose, ^18^*F*-2-Fluor-2-desoxy-D-glucose Positron Emission Tomography/Computed Tomography FDG-PET/CT scans performed in clinical routine six months after initiation of ICI were evaluated according to RECIST 1.1.

All FDG-PET/CT scans used for this purpose were performed at the Department of Nuclear Medicine of the University Hospital Zurich, according to the department’s standard protocol. 

Based on the RECIST 1.1. evaluation of FDG-PET/CT scans performed six months after initiation of ICI patients were split up into two groups: patients with disease progression (progressive disease PD) versus patients with clinical benefit (complete response CR, partial response PR and stable disease SD).

- A Long-term response to ICI

The long-term response to ICI was defined by the overall survival OS (e.g., the time from first treatment to death or last follow-up) and progression-free survival PFS (e.g., the time from first treatment to disease progression or death). 

### 2.3. Statistical Analysis

All statistical computations were performed using R (version 3.3.3). For descriptive statistical analyses continuous variables were summarized as median, mean and range, while categorical variables as frequencies. Chi-square tests and *t*-tests were used for the calculation of sex-related differences regarding baseline characteristics, inflammatory response in peripheral blood, treatment toxicity and treatment response in patients with advanced melanoma treated with ICI. Kaplan–Meier survival curves were designed for men and women based on OS and PFS. Statistical significance was accepted at *p* < 0.050.

## 3. Results

### 3.1. Baseline Characteristics

In total, 103 patients with advanced melanoma (mean age 68 ± 13.83 years) were included, 29 women (mean age 60.41 ± 14.57 years) and 74 men (mean age 65.66 ± 13.34 years).

A total of 6 patients (3 women and 3 men) were stage III and 97 patients (26 women and 71 men) stage IV according to AJCC. In total, 86 patients (25 women and 61 men) were treated with dual checkpoint inhibition, while 17 patients (4 women and 13 men) with single checkpoint inhibition.

A significant difference between women and men was observed regarding the anatomical site of the primary tumor (*p* < 0.001). The primary tumor was located on a lower extremity in one out of three women and on the head/neck in one out of three men.

Furthermore, an additional significant trend (*p* < 0.001) could be highlighted from our results. While the superficial spreading (n = 25 men, 41%) and nodular (n = 22 men, 36%) melanoma subtypes represented together 77% (n = 47 men) of the cases in male population, women showed a more heterogenous distribution of melanoma subtypes with the superficial spreading (n = 9 women, 35%), nodular (n = 6 women, 23%), acral lentiginous (n = 5 women, 19%) and mucosal (3 women, 12%) melanoma subtypes being most frequent in female population.

**Figure 1:**
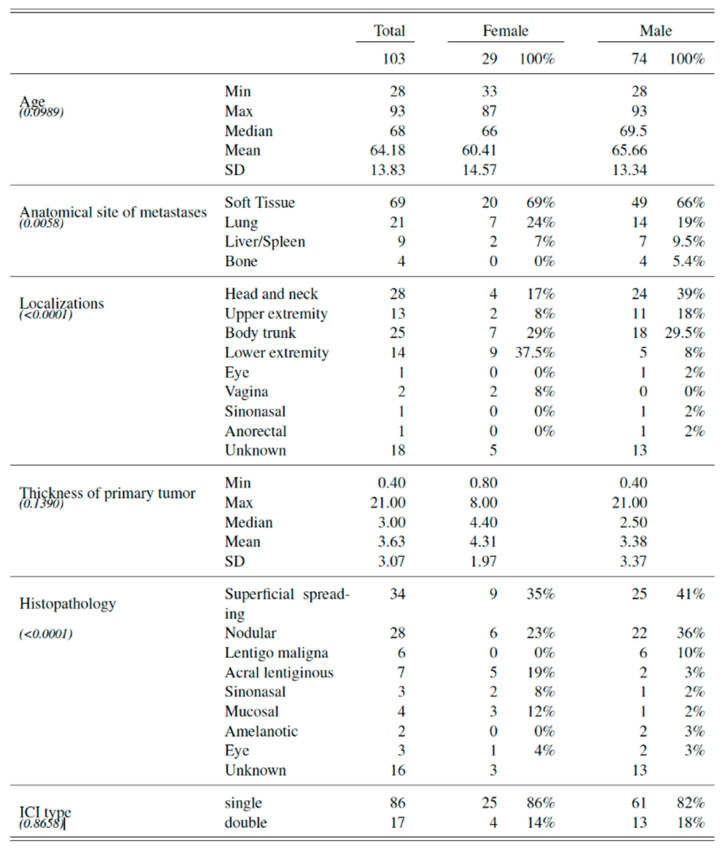
Baseline characteristics of all included female and male patients with advanced melanoma before initiation of immune checkpoint inhibition.

However, no significant difference between women and men was observed with regards to the thickness of the primary tumor (*p* = 0.139), patient age (*p* = 0.099) and anatomical sites of melanoma metastasis (*p* = 0.006). Figure 1.

### 3.2. Inflammatory Response to ICI in Peripheral Blood

Significant differences with regards to inflammatory parameters in peripheral blood between female and male patients with advanced melanoma could be outlined before treatment start and during the first months with immunotherapy.

First of all, women presented significantly lower counts of erythrocytes at baseline (*p* < 0.001), three (*p* < 0.001) and six months (*p* < 0.001) after initiation of ICI towards men.

Secondly, lower counts of monocytes were documented in women compared to men before treatment start (*p* < 0.001) and six months after initiating therapy (*p* < 0.001).

**Figure 2:**
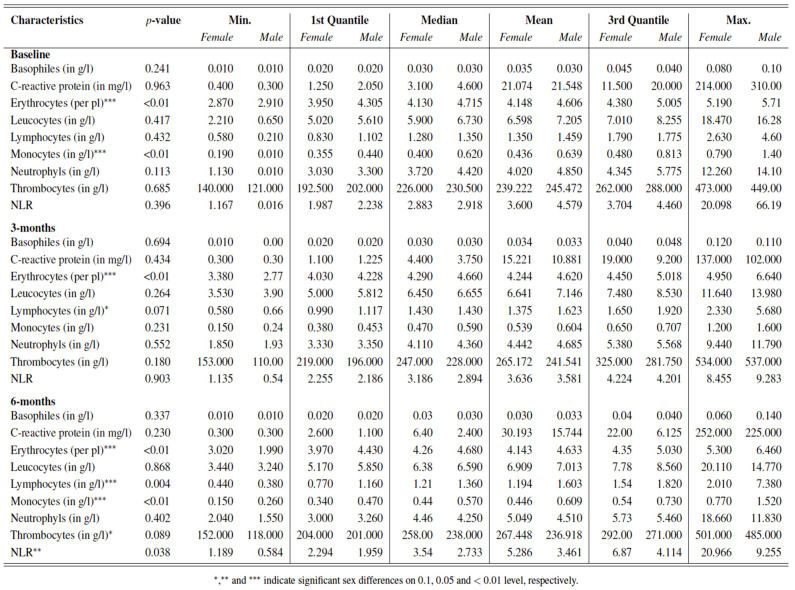
Blood parameters of all included female and male patients with advanced melanoma treated with ICI at baseline, and three and six months after treatment start.

Finally, most differences between women and men with regards to inflammatory parameters were observed six months after initiation of ICI with a higher median NLR (*p* = 0.038), lower counts of lymphocytes (*p* = 0.004) and thrombocytes (*p* = 0.089) in addition to lower counts of erythrocytes (*p* < 0.001) and monocytes (*p* < 0.001) in women towards men, as previously stated. Figure 2.

### 3.3. Treatment Toxicity

Significant and clinically relevant differences between male and female patients with advanced melanoma could be brought to light in context of toxicity due to immunotherapy.

First of all, immune-related adverse events were more frequent in women towards men (*p* = 0.013). Almost one out of two women experienced IRAEs (45%, n = 13 women) versus only one out of three men (32%, n = 24 men).

Secondly, a significant pattern could be drawn regarding the type of IRAEs between both groups (*p* < 0.001). Women were more likely to display endocrinological IRAEs, with thyroiditis being the most frequent adverse event (50% of all IRAEs in women, n = 9 women), followed by hypophysitis (17% of all IRAEs in women, n = 3 women). Interestingly IRAEs of the gastrointestinal tract were the most frequent ones in men (28%, n = 8 men), equally followed by disorders of the skin and thyroid gland (respectively 21%, n = 6 men) 

**Figure 3:**
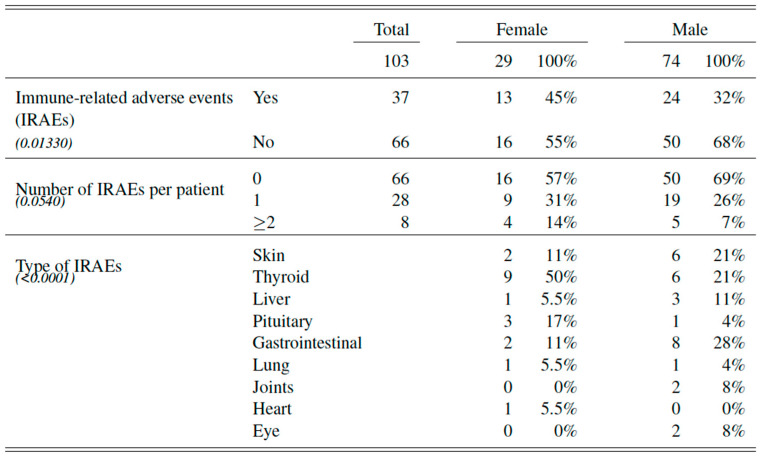
Immune-related adverse events in male and female patients with advanced melanoma treated with ICI.

Finally, no significant difference was seen in the number of IRAEs per patient between women and men (*p* = 0.054). Figure 3.

**Figure 4:**
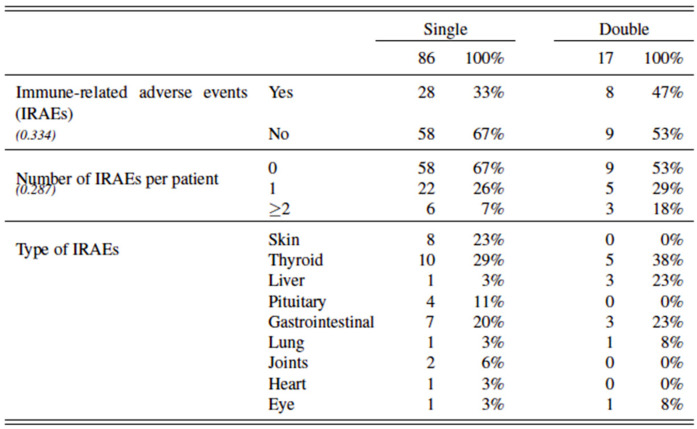
Immune-related adverse events in all included patients with advanced melanoma treated with single versus double ICI.

In addition, we also investigated whether treatment with double ICI led to more treatment toxicity, e.g., IRAEs, than single ICI. However, no significant differences were noted between both groups with regards to the onset, or number or the type of IRAE. Figure 4.

### 3.4. Patient Response to ICI

#### 3.4.1. Short-Term Response to ICI

**Figure 5:**
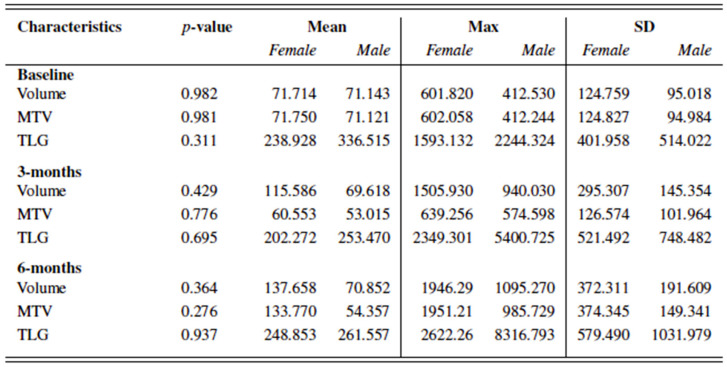
Total tumor load (volume in ml) and total metabolic tumor load (metabolic tumor volume MTV and total lesion glycolysis TLG) at baseline, and three and six months after initiation of ICI in female and male patients with advanced melanoma.

No significant differences were seen in the total tumor load and total metabolic tumor load on FDG-PET/CT scans between male and female patients with advanced melanoma before treatment, and three and six months after initiation of ICI. Figure 5.

**Figure 6:**
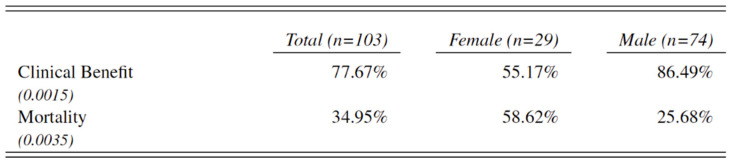
Clinical benefit in all included female and male patients with metastatic melanoma treated with ICI. Clinical benefit being defined as no disease progression displayed on FDG-PET/CT scan six months after initiation of ICI.

However, after six months of immunotherapy a vast majority of men benefitted from treatment (n = 64 men, 86.49%), while nearly one out of two women (n = 13, 44.83%) showed progressive disease under treatment (*p* = 0.001). Figure 6.

#### 3.4.2. Long-Term Response to ICI

We observed as long-term response to immunotherapy in female patients with advanced melanoma a mean overall survival OS of 590.24 ± 272.56 days and progression free survival PFS of 200.17 ± 234.74 days, while OS and PFS were significantly better in male patients with advanced melanoma, respectively, 843.62 ± 505.53 days (*p* = 0.001) and 440.61 ± 460.60 days (*p* < 0.001).

**Figure 7:**
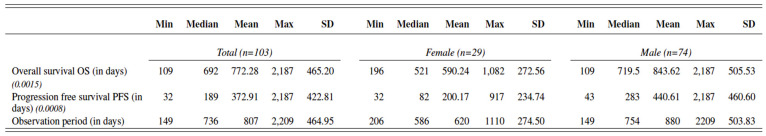
Overall survival OS and progression free survival PFS of female and male patients with advanced melanoma treated with ICI.

**Figure 8:**
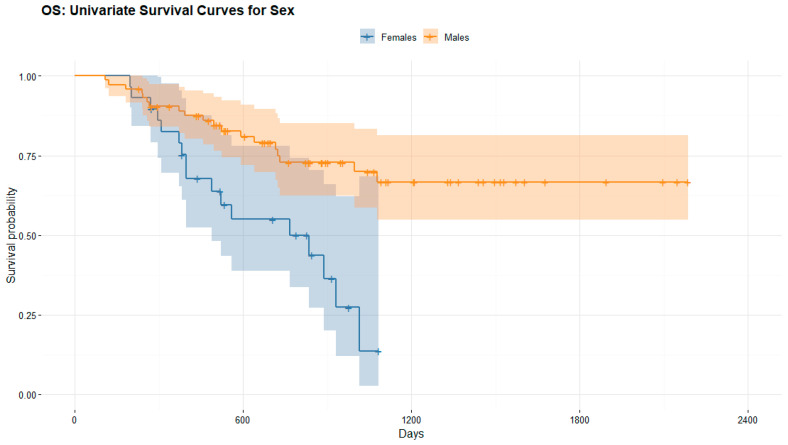
Kaplan–Meier survival curve stratified by overall survival OS (in days) in men (orange) and women (blue) with advanced melanoma treated with immunotherapy. The female curve ends earlier than the male curve due to a shorter follow-up time (mean 620 days vs. 880 days for men), as displayed in Figure 7.

**Figure 9:**
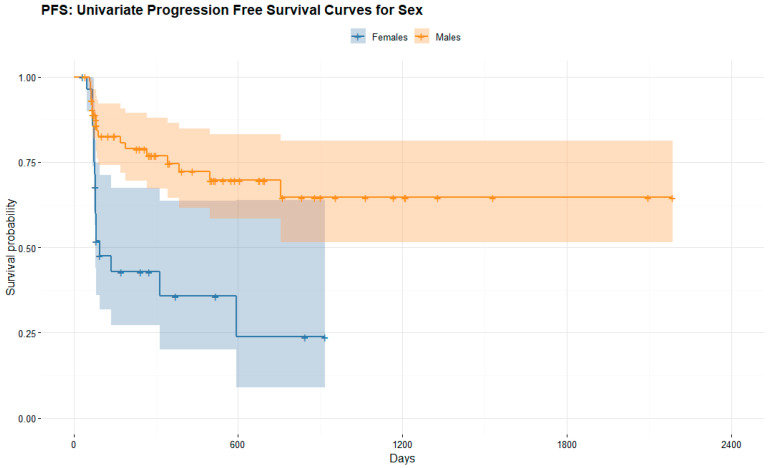
Kaplan–Meier survival curve stratified by progression free survival PFS (in days) in men (orange) and women (blue) with advanced melanoma treated with immunotherapy. The female curve ends earlier than the male curve due to a shorter follow-up time (mean 620 days vs. 880 days for men), as displayed in Figure 7.

In light of these results, long-term response to ICI was significantly better in men than women. Figure 7, Figure 8 and Figure 9.

**Figure 10:**
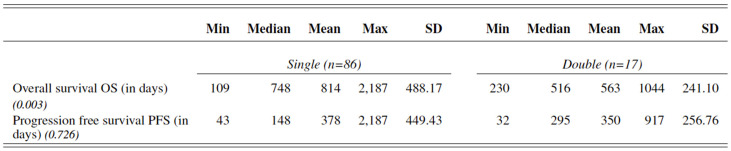
Overall survival OS and progression free survival PFS of female and male patients with advanced melanoma treated with single vs. double ICI.

In addition, we observed a significant improved overall survival in patients treated with single ICI towards patients treated double ICI (*p* = 0.003). Figure 10.

Finally, every fourth man (25,68%, n = 19) died during the course of the disease, while every second woman (58.62%, n = 17) did not survive. In knowledge of these results, mortality appeared significantly different between men and women (*p* = 0.001).

## 4. Discussion

In knowledge of the recent literature, we aimed to investigate whether higher immune activation in peripheral blood promotes the onset of IRAEs and a favorable short- and long-term outcome after ICI in men and women with advanced melanoma.

Applying our inclusion criteria, we obtained a male-dominated cohort with a mean age of 68 ± 13.83 years suggesting older men had higher rates of advanced melanoma in accordance with recent literature. Olsen et al. reported that women have higher rates of melanoma in early life, and men in later life [5]. After the age of 75 years, the incidence rate in men rises to three times the incidence rate in women according to Castro e Souza et al. [28].

Our results brought surprising and intriguing differences between male and female melanoma patients to light regarding immunotoxicity and immune profile in peripheral blood during immunotherapy. 

First of all, IRAEs were more frequent in women than men. Given the higher mortality in women after ICI, we investigated based on internal clinical records, whether the higher frequency of IRAEs led to higher rates of treatment discontinuation in women compared to men with advanced melanoma, which was not the case in our cohort. This might be explained by the type of side effects displayed. In fact, women were more likely to display not severe endocrinological IRAEs and men gastrointestinal IRAEs. 

Secondly, while we would have expected an overdriven immune response in women during ICI, as a hypothetical explanation for the frequent IRAEs, women, surprisingly, showed lower immune profile in peripheral blood at baseline, and three and six months after initiation of ICI. Significant differences with regards to inflammatory parameters in peripheral blood between male and female patients with advanced melanoma could be highlighted, particularly six months after initiation of ICI. At baseline, significant lower counts of erythrocytes and monocytes were displayed in women towards men. Interestingly, three months after initiating ICI, a short-term increase in monocytes could be seen in all patients with no significant results in men and women, presumably displaying an systemic immune response to immunotherapies. At that time, three months after treatment start, women displayed significant lower counts of erythrocytes and lymphocytes. Three months later, a new decrease in monocytes counts in all patients could be observed with significant differences between men and women, as well as similar values as before treatment. Women showed at this stage of the treatment significantly lower counts of lymphocytes, lower counts of monocytes and higher median NLR than men six months after initiating ICI. Nakamura et al. reported in 2016 that higher counts of lymphocytes and lower counts of neutrophils during treatment with immunotherapy were early markers of treatment response associated with better overall survival [23]. In this context, the neutrophils-to-lymphocytes ratio, NLR, has been described by various recent studies as promising biomarker for treatment response in melanoma patients treated with immunotherapy, with high NLR being associated with poor overall survival [20].

Our results suggested that immune activation in peripheral blood might not be coupled with the onset of IRAEs. However, a few limitations with regards to factors interfering with blood immune activation should be addressed. Although some of the included women were still at a childbearing age, the median and mean age of all 29 women included was 66 and 60.4 years, respectively. Therefore, the interaction between female sex hormones and the PD-1 and PD-L1 pathway and the effect of bleeding (potentially leading to anemia) during treatment may have been limited in this cohort. Moreover, although acute infections in men and women could be ruled out on FDG-PET/CT at baseline, three and six months after treatment start, other factors (such as previous systemic treatment and drug history) might also have influenced the immune activation in peripheral blood in men and women at baseline and after initiating ICI, particularly since the majority of the patients (N = 78) were pretreated.

Interestingly, men with advanced melanoma showed a significantly better response to immunotherapy in short- and long-term than women. In fact, 86.49% of men showed clinical benefit six months after ICI versus 55.17% of women. In the long-term, every fourth man died during the course of the disease, while every second woman did not survive. Wang et al. summarized in a review published in 2019 the results of recent investigations with regards to sex differences in immunotherapy efficacity. According to the authors, the hot immune tumor environment in women would cause low antigenicity of tumor cells, leading to less efficacity of immunotherapies, as opposed to men with cold immune tumor environment and high antigenicity of tumor cells leading to a better efficacity of immunotherapies [8]. In our cohort, men showed, on the contrary, higher immune activation in peripheral blood than women at baseline, and three and six months after treatment start with significantly improved outcome in short- and long-term compared to women. In light of our innovative results, higher immune environment in peripheral blood before and after initiation ICI might be linked to favorable treatment response during and after ICI in favor of men and decoupled from the onset of IRAEs, as the second innovative highlight of our investigations.

In addition, further significant differences between female and male melanoma patients treated with ICI were observed regarding their baseline characteristics. The primary tumor was located on a lower extremity in one out of three women and on the head/neck in one out of three men. Lower extremities for women and head/neck for men have been reported as anatomical site of predilection for melanoma primary tumor by recently published investigations [5,10,28].

Furthermore, an additional significant trend could be highlighted from our results. While the superficial spreading and nodular melanoma subtypes represented together 77% of the cases in male population, women showed a more heterogenous distribution of melanoma subtypes with the superficial spreading, nodular, acral lentiginous and mucosal melanoma subtypes being most frequent in female population. Pala et al. recently highlighted the role played by melanoma histological subtypes in response to immunotherapy. Their results published in 2022 displayed better overall response rates in patients with nodular melanoma versus patients with superficial spreading melanoma [29]. In our cohort, men showed higher rates of nodular melanoma compared to women. Furthermore, the more heterogenous distribution of melanoma subtypes in women included histological subtypes with poor prognosis, such as sinonasal melanoma [30] and mucosal melanoma [31], which might also have influenced the worse outcome, particularly the higher mortality experienced by women towards men in our cohort.

In conclusion, three major observations with two innovative insights can be highlighted in light of our investigations. First of all, as well established, IRAEs were more frequent in women towards men. Secondly, the onset of IRAEs was surprisingly not coupled with a higher immune activation in peripheral blood in women. On the contrary, women showed during the first six months of ICI lower blood immune activation towards men. Finally, the higher immune activation in peripheral blood observed in men was interestingly associated with a significantly better outcome in men than women. Given the significantly higher immunotoxicity and worse outcome experienced by women compared to men, the use of ICI should be chosen carefully in women with advanced melanoma. 

Here, we provided evidence for significant differences between men and women with advanced melanoma treated with ICI with regards to their baseline characteristics, inflammatory response in peripheral blood, treatment toxicity and response to treatment. 

The limitations of our investigations are the retrospective approach and the size of our cohort, particularly the lower number of female patients than male patients applying our inclusion criteria. Both limitations could be overcome by further prospective investigations on the underlying biology of these sex-related differences with larger proportion of women are needed in the future.

## 5. Conclusions

Men with advanced melanoma showed a significantly better response to immunotherapy in short- and long-term towards women. Higher immune activation in peripheral blood before and after initiation ICI might be linked to favorable treatment response during and after ICI in favor of men and decoupled from the onset of IRAEs. Given the significantly higher immunotoxicity and worse outcome experienced by women compared to men the use of ICI should be chosen carefully in women with advanced melanoma.

## Data Availability

All reviewed imaging modalities and clinical data were assessed during clinical routine. Patient data are stored in local archiving system.

## Abbreviations

ICI	Immune Checkpoint Inhibitor
CTLA-4	Cytotoxic T-Lymphocyte-associated Antigen-4
PD-1	Programmed cell death protein-1
IRAEs	Immune-Related Adverse Events
GCP	Good Clinical Practice
AJCC	American Joint Committee on Cancer
NLR	Neutrophils-to-Lymphocytes Ratio
FDG-PET/CT^18^*F*-2-Fluor-2-desoxy-D-glucose Positron Emission Tomography/Computed Tomography	
RECIST 1.1.	Response Evaluation Criteria in Solid Tumors version 1.1.
PD	Progressive Disease
CB	Clinical Benefit
CR	Complete Response
PR	Partial Response
SD	Stable Disease
OS	Overall Survival
PFS	Progression-free survival PFS
MTV	Metabolic Tumor Volume
TLG	Total Lesion Glycolysis
